# Prognostic value of lymph nodes ratio in patients with stage III ovarian clear cell carcinoma: A retrospective study of patients in Southwest China

**DOI:** 10.7150/jca.29896

**Published:** 2019-08-19

**Authors:** Dan Nie, Xiguang Mao, Zhengyu Li

**Affiliations:** 1 Department of Obstetrics and Gynecology, Key Laboratory of Birth Defects and Related Diseases of Women and Children, Ministry of Education, West China Second University Hospital, Sichuan University, Chengdu,610041, People's Republic of China.; 2 Department of Obstetrics and Gynecology, The affiliated hospital of Southwest Medical University, Luzhou,646000, People's Republic of China.

**Keywords:** ovarian clear cell carcinoma, lymph node ratio, survival

## Abstract

**Background:** Ovarian clear cell carcinoma (OCCC) has a worse prognosis compared to other histological subtypes. Although the survival effect of lymph nodes ratio (LNR) on ovarian carcinoma have been elucidated in several studies, the prognostic effect of LNR in OCCC has not been separately studied. This study aimed to investigate the prognostic significance of LNR in FIGO stage III OCCC.

**Methods:** Patients with FIGO stage III OCCC who underwent primary cytoreductive surgery and systematic lymphadenectomy from January 2008 to June 2014 in two independent hospitals were retrospectively reviewed. Two independent patients cohorts were used to investigate the survival impact of LNR by using Kaplan-Meier and Cox regression proportional hazard method.

**Results:** In training cohort, the 5-year progression-free survival (PFS) rates was 32.4% for patients with LNR ≤ 25%, and 19.8% for patients with LNR > 25%, respectively (p = 0.017). The 5-year overall survival (OS) rates was 41.3% for patients with LNR ≤ 25%, and 25.8% for patients with LNR > 25%, respectively (p = 0.003). In multivariate analysis, increased LNR was correlated with a poorer DFS (HR = 2.12 ,95% CI 1.32-3.41, p = 0.002) and OS (HR = 2.29, 95% CI 1.37-5.12, p = 0.001). These results were verified in a validation cohort.

**Conclusions:** LNR is an independent survival predictor in patients with FIGO stage III OCCC.

## Introduction

Epithelial ovarian cancer (EOC) is a group of diseases with distinct clinical and histopathological features.^1^, ^2^ Additionally, the incidence rate of lymph nodes (LNs) metastasis also differ in different EOC histological types and grades.^3^-^5^ The standard management for advanced EOC is cytoreductive surgery and systematic lymphadenectomy followed by platinum-based and taxane-based chemotherapy 6, 7. Currently, the prognostic and therapeutic significance of systematic lymphadenectomy in EOC remains controversial^8^. However, LNs metastasis predicts poor survival in EOC patients has been well confirmed 9, 10.

Lymph node ratio (LNR), defined as the ratio of the number of metastatic lymph nodes (MLNs) to the number of resected lymph nodes (RLNs)^11^, has been proved as an independent prognostic predictor in several malignancies including nonsmall cell lung cancer^12^, breast cancer^13^, cervical cancer^14^, endometrial cancer^15^, and EOC 8, 16-19. However, the previous studies investigated the survival predictive value of LNR in all histologic subtypes of EOC and did not validate their results in another independent population.^8^, ^16^-^19^. In addition, the prognostic impact of LNR in ovarian clear cell carcinoma (OCCC), which accounts for approximately 5% to 25% of primary EOC, has not been separately clarified. 20 Furthermore, advanced OCCC, prone to chemo-resistant, has decreased survival compared with other histologic subtypes of EOC.^2^, ^20^, ^21^ Moreover, there has no previous study assessed the prognostic role of LNR in Chinese population with advanced OCCC.

In the current study, we aimed to investigate the prognostic significance of LNR in two independent cohorts of Chinese patients with FIGO stage Ⅲ OCCC.

## Methods

### Patients selection

OCCC patients who underwent surgical staging and lymphadenectomy in West China Second University Hospital, Sichuan University, and The Affiliated Hospital of Southwest Medical University from January 2008 to June 2014 were reviewed. Patients were enrolled into study according to the following inclusion criteria: (1) patients with a diagnosis of FIGO stage III; (2) patients underwent total hysterectomy, salpingo-oophorectomy, pelvic and para-aortic lymphadenectomy, omentectomy, and resected any suspicious and/or enlarged disease; (3) LNs metastasis positive; (4) no residual disease or residual disease <1cm. Patients who had received neoadjuvant chemotherapy and interval debulking surgery were excluded from this study. The patient's age at diagnosis, clinicopathologic characters, treatment, and survival status was collected from the patients' medical records and clinical follow-up visits. 35 LNs was used as the cut-off value of systematic lymphadenectomy according to previous literature reported 21. To investigate the survival impact of LNR, LNs positive patients were assigned into two groups according to reported 8: LNR1 (LNR≤25%), and LNR2 (>25%). The primary outcome was overall survival (OS) and progression-free survival (PFS). Patients from January 2008 to December 2012 was arranged in the training cohort, while patients from January 2013 to June 2014 was arranged in the validation cohort.

### Statistical analysis

Correlations between categorical covariates were analyzed using chi-square test or Fisher's exact test. The PFS and OS curves were generated using Kaplan-Meier method and compared using log-rank test. Cox proportional hazard model was performed to assess the association between LNR and PFS and OS. SPSS™, version 20.0 (SPSS Inc, Chicago, IL, USA) was used to performing the statistical analyses. *P* values < 0.05 were considered statistically significant.

## Results

Patient baseline data in the training cohort are summarised in Table 1. The median patient age was 56 years old (30-89). The median follow up time was 40 months (1-119). The median number of RLNs was 46 (30-92). LN metastatic patients including 72 patients with both pelvic and para-aortic MLNs (40.4%), 79 patients only have pelvic MLNs (44.4%), and 27 patients only have para-aortic MLNs (15.2%). The median number of total MLNs was 5 (1-69). The median number of pelvic MLNs and para-aortic MLNs was 4 (0-47) and 3 (0-22), respectively. There were no significant differences between LNR and patient clinicopathologic characteristics in the training cohort (Table 2).

**Figure 1:**
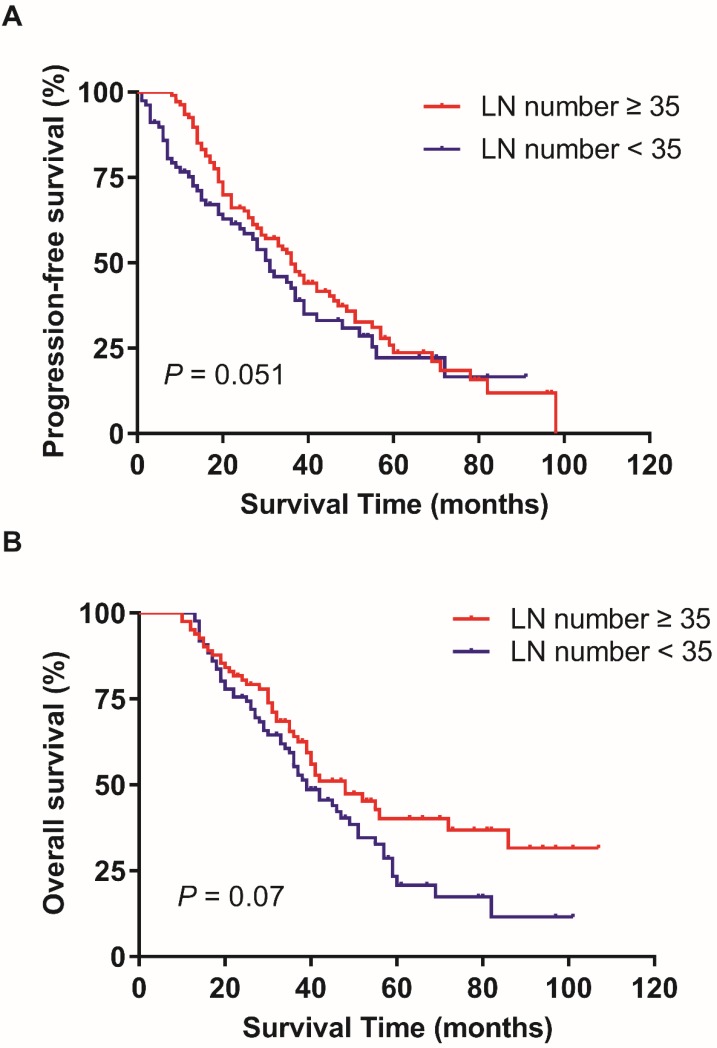
Effect of the number of resected lymph nodes on progression-free survival (A) and overall survival (B). Kaplan-Meier.

**Figure 2:**
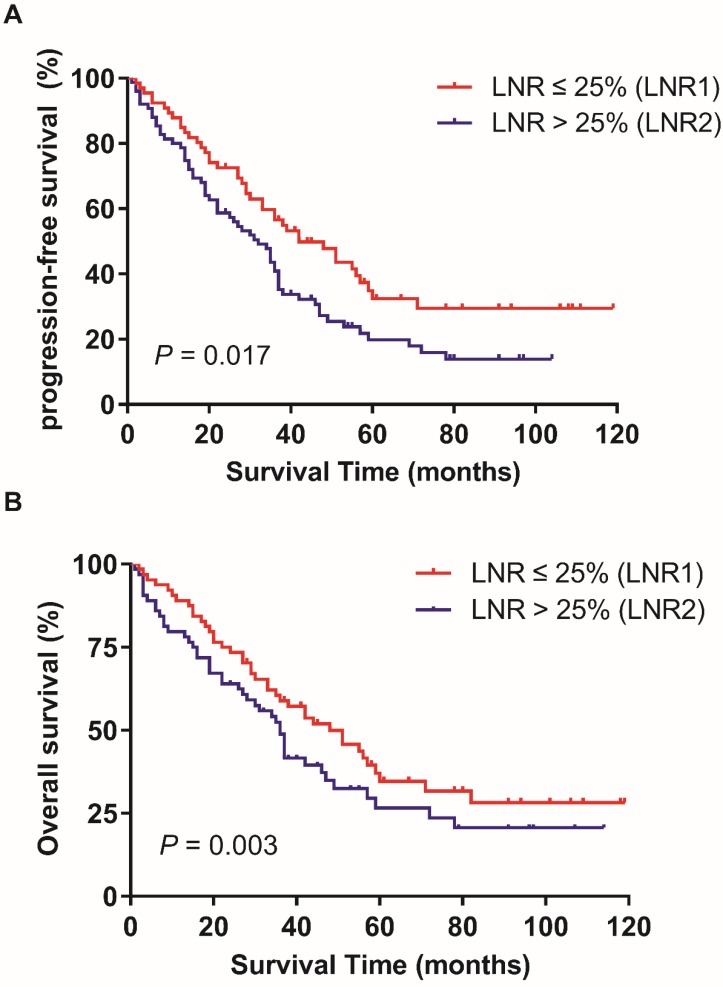
Effect of LNR (lymph node ratio) on progression-free survival (A) and overall survival (B) in training cohort. Kaplan-Meier.

In the training patients cohort, the PFS and OS has no statistically significant difference in patients with ≥ 35 RLNs and < 35 RLNs (p = 0.051, p = 0.07; Figure 1). The median LNR was 8.7% (1.9%-72.7%). The median LNR was 6.7% (1.9%-7.6%) for stage IIIA1, 6.5% (2.1%-7.8%) for stage IIIB, and 9.1% (1.9%-72.7%) for stage IIIC. The 5-year PFS rates in LNR1, LNR2 was 32.4%, and 19.8% respectively (p = 0.017; Figure 2A).The 5-year OS rates in LNR1, LNR2 was 41.3%, and 25.8% respectively (p = 0.003; Figure 2B).

Further Cox univariate analysis revealed FIGO stage and LNR was related to PFS and OS. However, the residual tumor size was related to PFS, but not OS (Table 3). In multivariate analysis, the LNR was an independent predictor of PFS and OS (Table 3). OCCC patients in LNR2 group (LNR>0.25) had an increased risk of relapse and mortality. The HR was 2.12 (95% CI 1.32-3.41) for PFS and 2.29 (95% CI 1.37-5.12) for OS (Table 3).

**Figure 3:**
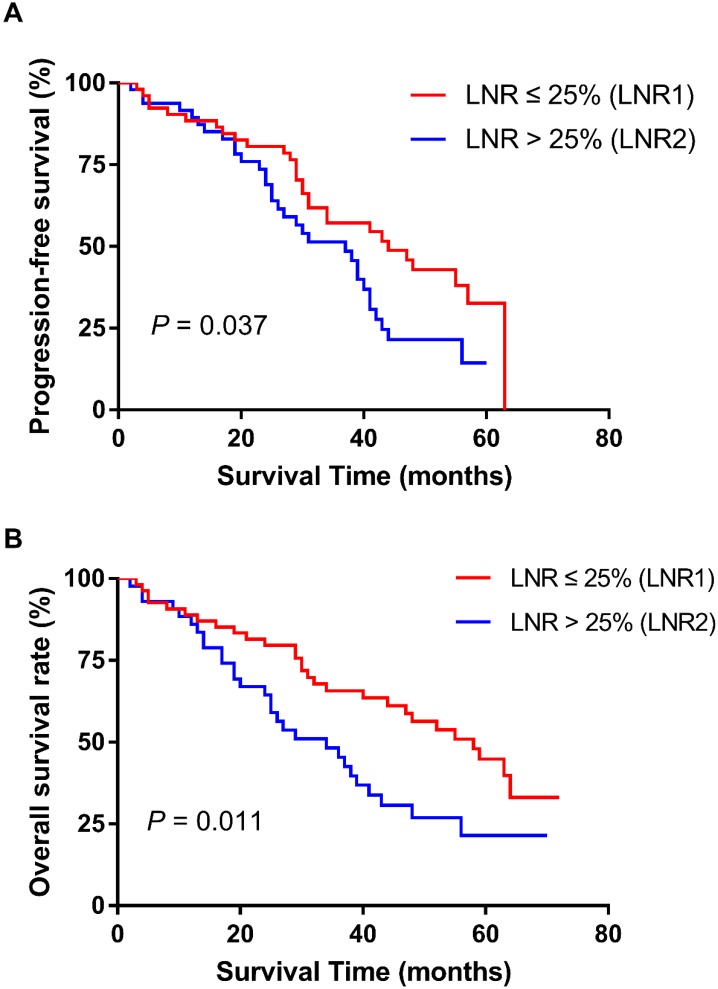
Effect of LNR (lymph node ratio) on progression-free survival (A) and overall survival (B) in validation cohort. Kaplan-Meier.

We further confirmed our results in the validation cohort. The patient baseline data and the association between the LNR and patient clinicopathologic characteristics also are showed in Table 1 and Table 2. The result indicated that the elevated LNR was correlated with worse PFS (p = 0.037) and OS (p = 0.011) (Figure 3). Univariate and multivariate analyses also proved the prognostic role of LNR (Table 4).

## Discussion

In the current study, we verified that LNR is an independent survival predictor for FIGO stage Ⅲ OCCC patients. Patients with elevated LNR (LNR>0.25) have a worse PFS and OS.

Although systematic lymphadenectomy is essential to establish stage of EOC, its therapeutic role in advanced ovarian cancer still controversial.^22^-^25^ Recently, randomized controlled studies revealed that patients with EOC did not gain a survival benefit from systematic lymphadenectomy.^22^, ^24^, ^26^ However, systematic lymphadenectomy might improve OCCC patients survival through remove of chemo-resistant metastatic LNs.^2^ Therefore, advanced OCCC patients might benefit from systematic lymphadenectomy.

The optimal lymphadenectomy cut-off value in OCCC patients has not been defined. Takei et al. showed that patients with ≥35 LNs removed have an improved recurrence-free survival.^21^ Pereira et al. study defined optimal lymphadenectomy cut-off value was at least obtain 15 pelvic LNs and 7 aortic LNs.^27^ However, the number of metastatic LNs is depended on many factors such as the surgeon and the pathologist's distinct experience in searching for positive LNs, the patients' anatomic variation, the extent of the tumor, and patients'age.^16^, ^17^ Hence, the limitation of use LNs status to predict survival might be addressed by using LNR.

The prognostic role of LNR has been discussed in advanced EOC. Ataseven et al. 8 found the 5-year OS rates were higher in patients with LNR ≤ 0.25 compared to patients with LNR > 0.25 (42.5% vs.18.0%). Ayhan et al. 17 focused on the FIGO stage III high-grade ovarian serous carcinoma (HGOSC), which also have a poor prognosis. They found the 5-year OS was decreased from 65.1% in LNR1(<10%) to 42.5% in LNR2(10%≤LNR<50%), and to 25.6% in LNR3(≥50%), LNR also was an independent survival predictor for OS. In the current study, we proved LNR was an independent predictor for decreased PFS and OS in FIGO stage Ⅲ OCCC patients. These results validated the feasibility of use LNR to predict prognosis in FIGO stage Ⅲ OCCC.

The LNR cut-off point used to assign patients to a lower or higher LNRs group has not been well defined. 8, 16-18 In this study, we used the LNR cut-off value described by Ataseven et al. 8. Further studies are needed to establish a standard LNR cut-off point. In addition, in the lymphadenectomy in ovarian neoplasms (LION) study, although 56% EOC patients had LNs micro-metastases, systematic lymphadenectomy offers no benefit to patients who underwent maximum or optimal cytoreduction and had clinically and radiologic negative lymph nodes.^28^ In our study, we defined optimal lymphadenectomy as the LION study described.^28^ The result indicated that LNR might an independent predictor for worse OS and PFS in FIGO stage Ⅲ OCCC patients with LNs metastasis. However, the resected LN number did not have survival effects in patients with OCCC.

Compared to previous studies, our study has several advantages. Above all, this is the first study investigating the prognostic role of LNR in OCCC based on Chinese population. Previous studies all based on European population 8, 17 or the SEER (Surveillance, Epidemiology, and End Results) database from the United States. 16, 18, 19 The postoperative adjuvant therapy information does not provide in SEER. Secondly, this study mainly focused on the prognostic value of LNR in OCCC, since OCCC has a poorer prognosis than other histological subtypes. Thirdly, previous studies did not validate their findings and conclusions using an independent validation cohort.^11^-^15^ In order to strengthen the credibility of our study, two independent patients cohorts were used to assess the prognostic role of LNR, and both patients cohort proved the prognostic value of LNR. However, our findings should be validated in future prospective study.

## Conclusions

LNR has a significant impact on PFS and OS and might be used as a predictor of survival in patients with advanced OCCC. However, these findings need to be verified in future prospective studies.

## Figures and Tables

**Table 1:** Clinical characteristics of the patients in training and validation cohort

Patients characteristic	Training cohort		Validation cohort
	N = 178 (%)		N = 87 (%)
**Follow-up (month)**	40 (1-119)		38 (2-72)
**Age (year)**	56 (30-89)		56 (27-85)
**Grade**			
2	47 (26.4)		9 (10.3)
3	131 (73.6)		78 (89.7)
**FIGO stage**			
IIIA1	39 (21.9)		3 (3.4)
IIIB	15 (8.4)		7 (8.1)
IIIC	124 (69.7)		77 (88.5)
**Peritoneal cytology**			
Positive	102 (57.3)		32 (36.8)
Negative	54 (30.3)		38 (43.6)
Not available	22 (12.4)		17 (19.6)
**Residual disease after surgery**			
0	82 (46.1)		36 (41.4)
1-10 mm	96 (53.9)		51 (58.6)
**Number of resected lymph nodes**			
Pelvic and para-aortic lymph nodes	46 (30-92)		34 (23-106)
Pelvic lymph nodes	32 (20-64)		21 (15-74)
Para-aortic lymph nodes	21 (10-31)		10 (8-32)
**Patients with lymph nodes metastasis**			
Only pelvic lymph nodes metastasis	79 (44.4)		52 (59.8)
Only para-aortic lymph nodes metastasis	27 (15.2)		7 (8)
Both pelvic and para-aortic lymph nodes metastasis	72 (40.4)		28 (32.2)
**Number of metastatic lymph nodes**			
Pelvic lymph nodes	4 (0-47)		2 (0-35)
Para-aortic lymph nodes	3 (0-22)		3 (0-29)
Pelvic lymph nodes and para-aortic lymph nodes	5 (1-69)		5 (1-64)
**Lymph node ratio (%)**			
≤25	106 (59.6)		49 (56.3)
>25	72 (40.4)		38 (43.7)
**Adjuvant chemotherapy**			
Yes	150 (84.3)		78 (89.7)
No	28 (15.7)		9 (10.3)
Status			
Alive	60 (33.7)		41 (47.1)
Dead	118 (66.3)		46 (52.9)

**Table 2:** Correlation between LNR and clinicopathological characteristics in the training and validation cohort

Patient characteristics		Training cohort							Validation cohort					
		LNR ≤ 25%			LNR > 25%		*P*		LNR ≤ 25%			LNR > 25%		*P*
		N = 106	%		N = 72	%			N = 49	%		N = 38	%	
**Median age, years (range)**		57 (30-83)			56 (33-80)		0.594		54 (29-85)			56 (27-80)		0.574
**Grade**														
2		31	29.2		16	22.2	0.387		6	12.2		3	7.9	0.509
3		75	70.8		56	77.8			43	87.8		35	92.1	
**FIGO stage**														
IIIA1		20	18.9		19	26.4	0.029		2	4.1		1	2.6	0.742
IIIB		5	4.7		10	13.9			4	8.2		3	7.9	
IIIC		81	76.4		43	59.7			43	87.7		34	89.5	
**Residual Disease**														
0		48	45.3		34	47.2	0.799		19	38.8		17	44.7	0.576
1-10 mm		58	54.7		38	52.8			30	61.2		21	55.3	
Peritoneal cytology^*^														
Negative		35	33		19	26.4	0.312		24	58.5		14	48.3	0.396
Positive		49	46.2		53	73.6			17	41.5		15	51.7	
**Adjuvant chemotherapy**														
Yes		89	84		61	84.7	0.891		45	91.8		33	86.8	0.448
No		17	16		11	15.3			4	8.2		5	13.2	
														

^*^The patients with peritoneal cytology status not available were not included.

**Table 3:** Univariate and multivariate analyses of patients for OS and DFS in training cohort

Characteristic	OS								DFS						
	Univariate analyses				Multivariate analyses				Univariate analyses				Multivariate analyses		
	HR	95% CI	*P*		HR	95% CI	*P*		HR	95% CI	*P*		HR	95% CI	*P*
**Grade**															
2	1								1						
3	1.24	0.78-2.13	0.314						1.39	0.87-2.08	0.746				
**Peritoneal cytology**															
Negative	1								1						
Positive	1.19	0.63-2.22	0.592						1.37	0.81-2.32	0.235				
**Residual disease**															
0	1								1				1		
1-10 mm	1.43	0.63-3.25	0.393						1.31	1.03-2.76	0.001		1.3	1.04-2.43	0.023
**FIGO stage**															
IIIA1 vs. IIIB	1.73	1.12-2.62	0.003		1.71	0.45-3.22	0.242		1.42	1.01-1.73	0.016		1.23	0.63-1.68	0.55
IIIA1 vs. IIIC	2.91	1.94-8.94	0.014		2.83	0.78-7.42	0.381		1.66	1.16-2.18	0.025		1.46	0.77-1.96	0.319
**Lymph node ratio (LNR)**															
≤25%	1				1				1				1		
>25%	2.92	1.45-4.89	<0.001		2.29	1.37-5.12	0.001		2.52	1.56-4.07	<0.001		2.12	1.32-3.41	0.002

**Table 4:** Univariate and multivariate analyses of patients for OS and DFS in validation cohort

Characteristic	OS								DFS						
	Univariate analyses				Multivariate analyses				Univariate analyses				Multivariate analyses		
	HR	95% CI	*P*		HR	95% CI	*P*		HR	95% CI	*P*		HR	95% CI	*P*
**Grade**															
2	1								1						
3	1.03	0.68-1.4	0.876						1.07	0.81-1.73	0.373				
**Peritoneal cytology**															
Negative	1								1						
Positive	1.31	0.91-1.88	0.149						1.15	0.79-1.68	0.471				
**Residual disease**															
0	1				1				1				1		
1-10 mm	1.44	1.13-1.83	0.013		1.67	1.19-2.34	0.038		1.81	1.48-2.20	0.001		1.90	1.39-3.74	0.03
**FIGO stage**															
IIIA1 vs. IIIB	1.2	0.65-2.23	0.599						1.066	0.66-1.71	0.814				
IIIA1 vs. IIIC	1.39	0.78-1.42	0.265						1.13	0.71-1.8	0.602				
**Lymph node ratio (LNR)**															
≤25%	1				1				1				1		
>25%	3.35	1.87-5.98	<0.001		2.80	1.97-3.96	0.001		1.99	1.31-3.01	<0.001		1.96	1.44-2.68	<0.001
